# Metabolomic Analysis of Specific Metabolites in *Codonopsis pilosula* Soil Under Different Stubble Conditions

**DOI:** 10.3390/molecules29225333

**Published:** 2024-11-13

**Authors:** Fengbin Xu, Daiyu Qiu, Yurong Hu, Xianxian Chen, Zhonghu Li, Qian Li

**Affiliations:** 1 State Key Laboratory of Aridland Crop Science, Agronomy College, Gansu Agricultural University, Lanzhou 730070, China; 1073323020263@st.gsau.edu.cn (F.X.); 1073323020261@st.gsau.edu.cn (Y.H.); 1073323020264@st.gsau.edu.cn (X.C.); liqian1984@gsau.edu.cn (Q.L.); 2 Key Laboratory of Resource Biology and Biotechnology in Western China, Ministry of Education, Life Sciences College, Northwest University, Xi’an 710069, China; lizhonghu@nwu.edu.cn

**Keywords:** *Codonopsis pilosula*, nontargeted metabolomics, continuous cropping, rotation, rhizosphere soil metabolites

## Abstract

To investigate the soil-specific metabolites of *Codonopsis pilosula* under different stubble management practices, this study analyzed differentially abundant metabolites in the rhizosphere soils of rotational (DS) and continuous (LS) cropping systems via liquid chromatography–tandem mass spectrometry (LC–MS/MS)-based metabolomic approaches. The results revealed that 66 metabolites, including amino acids and their derivatives, nucleic acids, alcohols, organic acids, amines, fatty acids, purines, and sugars, were significantly different (*p* < 0.05) between the DS and LS groups. Under continuous cropping, the levels of amines, fatty acids, organic acids, and sugars in the rhizosphere soil were significantly greater (*p* < 0.05) than those under rotational cropping, whereas the levels of amino acids and their derivatives, nucleic acids, and purines and pyrimidines were significantly lower (*p* < 0.05). KEGG pathway enrichment analysis revealed that these differentially abundant metabolites were enriched in metabolic pathways such as amino acid metabolism (e.g., alanine, aspartate, and glutamate metabolism), carbon metabolism, the cAMP signaling pathway, ABC transporter proteins, phenylalanine metabolism, and the biosynthesis of plant secondary metabolites. These metabolic pathways were involved in osmoregulation, energy supply, and resilience in plants. In conclusion, inter-root soil metabolites in rotational and continuous cropping of *Codonopsis pilosula* were able to influence soil physicochemical properties and microbial populations by participating in various biological processes.

## 1. Introduction

*Codonopsis pilosula* was first recorded in the Qing Dynasty text *Ben Cao Cong Xin*. The official varieties included in the 2020 edition of the *Pharmacopoeia of the People’s Republic of China* are the dried roots of the perennial herbaceous plants of the Campanulaceae family: *Codonopsis pilosula* (Franch.) Nannf., *Codonopsis pilosula* Nannf. var. *modesta* (Nannf.) L.T. Shen, and *Codonopsis tangshen Oliv* [1]. It has a sweet taste and neutral properties, and it benefits the spleen and lung meridians. It is known for strengthening the spleen and lungs, nourishing the blood, and promoting the generation of body fluids. It is commonly used to treat conditions such as spleen and lung Qi deficiency, weak cough, chronic asthma, blood deficiency, and thirst due to fluid depletion [2]. Owing to its diverse medicinal functions, many scholars believe that the efficacy of *Codonopsis pilosula* is comparable to that of ginseng [3,4]. The chemical composition of *Codonopsis pilosula* is complex and diverse. Researchers, both domestic and international, have isolated and identified over 230 chemical constituents from *Codonopsis pilosula*, including active components such as polysaccharides, polyacetylenes, alkaloids, lignans, flavonoids, and terpenes [5,6]. Modern pharmacological studies have shown that *Codonopsis pilosula* possesses multiple pharmacological effects, including regulation of the spleen and stomach, blood sugar and lipids, enhancement of the immune function, antioxidation, reinforcement of the hematopoietic function, and antitumor activities. It can be used to treat cardiovascular diseases and various chronic spleen and stomach disorders, and it has significant inhibitory effects on various bacteria and viruses [7,8,9].

Continuous cropping refers to the repeated cultivation of the same or closely related crop species on the same land over successive years. Continuous cropping obstacles, however, occur in this practice, where despite professional growth and cultivation management, issues such as weak plant growth, low yield, uneven quality, and frequent pests and diseases inevitably arise [10,11,12]. Currently, in China, continuous cropping obstacles affect various crops, including grains, oilseeds, vegetables, fruits, and major medicinal plants, leading to significant declines in yield and quality [13,14]. For example, continuous cropping of perennial asparagus can significantly reduce yield and inhibit seed germination and seedling growth in the following year [15]. This phenomenon is even more pronounced in medicinal plants. Reports indicate that more than 70% of root-type medicinal plants suffer from continuous cropping obstacles [16]. *Panax ginseng*, *Panax notoginseng*, and *Panax quinquefolius* L. are particularly sensitive to continuous cropping. The survival rate of ginseng and American ginseng seedlings decreases to approximately 30% after one year of continuous cropping [17,18]. As the duration of continuous cropping increases, the activity of beneficial soil enzymes significantly decreases, often leading to nutrient imbalances in plants [19]. Owing to its regional specificity and unique characteristics, *Panax notoginseng* is highly sensitive to continuous cropping. It is generally believed that land used to grow *Panax notoginseng* should not be replanted with the same crop for ten years. Continuous cropping also exacerbates the wilt rate of *Salvia miltiorrhiza*, severely hindering plant growth and the accumulation of active constituents. *Codonopsis pilosula* is also sensitive to continuous cropping and typically requires a crop rotation cycle of 3–4 years before normal cultivation can resume [20].

Crop rotation is a traditional agricultural practice commonly used to reduce and avoid the occurrence of continuous cropping obstacles [21,22,23]. By alternating the types of crops planted, rotation prevents excessive depletion of soil nutrients and the accumulation of specific pests and diseases associated with long-term monocropping [24]. Since different crops have varying root distributions, nutrient requirements, and growth cycles, crop rotation ensures a more balanced utilization of soil nutrients. For example, leguminous crops have nitrogen-fixing capabilities, and rotating them with other crops helps enrich the soil nitrogen content and improve the soil fertility structure [25]. Crop rotation also mitigates the incidence of pests and diseases in the soil. Each pest and disease has specific host plants; changing the crop interrupts the suitable living conditions for pests and diseases, thereby inhibiting their reproduction and spread. For example, long-term cultivation of a particular vegetable might lead to the proliferation of specific pests. Rotating to nonhost crops can interrupt the life cycle of these pests, thereby reducing their adverse impact [26]. Additionally, crop rotation can increase the diversity and richness of soil microorganisms, promoting healthier plant growth [27,28,29].

Metabolites in soil play a crucial role in the soil ecosystem [30]. Generally, metabolites in soil include sugars, amino acids, organic acids, phenols, alkaloids, etc., [31,32,33]. They can directly affect the physical and chemical properties of soil. For example, organic acids can promote minerals to release nutrients, improve nutrient availability, and at the same time affect soil pH and create an environment suitable for plant growth [34,35]. They can also provide carbon sources and energy for soil microorganisms, drive changes in microbial community structure, and indirectly affect plant growth [36]. In addition, some metabolites can directly provide nutrition for plants. For example, amino acids participate in protein synthesis, and when plants are under environmental stress, some metabolites help enhance the stress resistance of plants. These metabolites play a key role in soil nutrient cycling, biological activity regulation, and ecosystem balance maintenance [37]. Li’s research found that some beneficial metabolites, such as organic acids and lipids, are helpful for nutrient availability and microbial activity. Their content will gradually decrease with the increase of continuous cropping years of *Codonopsis pilosula* [38]. Yang et al. identified 47 differential metabolites from the rhizosphere soil of *Codonopsis pilosula* in different continuous cropping years, including 16 lipids, 10 acids, 6 sugars, 5 other substances, 4 esters, 4 phenols, and 1 amine [39].

Untargeted metabolomics offers significant advantages in the analysis of soil-specific metabolites. It can comprehensively and unbiasedly detect as many metabolites as possible in the soil, avoiding the need for presetting and screening specific metabolites. This has led to the discovery of unknown and potentially significant specific metabolites [40]. This method can provide a more complete and accurate metabolic profile of the soil, reflecting the complex metabolic state of the soil ecosystem [41]. LC–MS/MS (liquid chromatography–tandem mass spectrometry) is widely used for analyzing soil-specific metabolites because of its high sensitivity, good selectivity, and strong separation capabilities [42,43]. It can separate and detect trace amounts of specific metabolites in complex soil matrices, effectively distinguish structurally similar compounds, and accurately identify target metabolites. Metabolomic methods can be used to analyze various metabolites of different natures simultaneously, thus providing comprehensive metabolite information [44,45,46]. Yuan et al. used LC–MS/MS to determine the metabolites in *Salvia miltiorrhiza* roots, investigating the primary metabolites responsible for cadmium resistance under varying levels of cadmium stress and revealing the metabolic response mechanisms of Salvia miltiorrhiza to cadmium stress [47]. Li et al. utilized untargeted metabolomics based on LC–MS to analyze rice samples from different regions, distinguishing the metabolic differences among various rice varieties [48]. Fu et al. systematically studied the composition, abundance, and metabolic pathways of metabolites in continuously cropped ramie via LC–MS and GC–MS via metabolomics, revealing significant changes in the levels of phenylpropanoids, fatty acids, amino acids, and other important metabolites [49]. However, few studies have reported the application of nontargeted metabolomics to explore the composition of metabolites in rhizosphere soil when *Codonopsis pilosula* is continuously cropped, and how rotation alleviates continuous cropping obstacles by changing the composition of metabolites in rhizosphere soil. This study employs LC–MS/MS to analyze specific metabolites in the rhizosphere soil of *Codonopsis pilosula* under continuous and rotational cropping regimes. This method can identify and quantify the specific metabolite components in the soil under different cropping regimes, elucidate the influence mechanisms of cropping systems on the composition and distribution of soil metabolites, and identify biomarkers causing continuous cropping obstacles. These findings provide a crucial basis for mechanistic studies of continuous cropping obstacles in *Codonopsis pilosula*, potentially alleviating such obstacles by regulating relevant metabolites and metabolic pathways, thereby promoting the healthy development of the *Codonopsis pilosula* industry.

## 2. Results and Discussion

### 2.1. Principal Component Analysis of the Soil Samples

**Figure 1:**
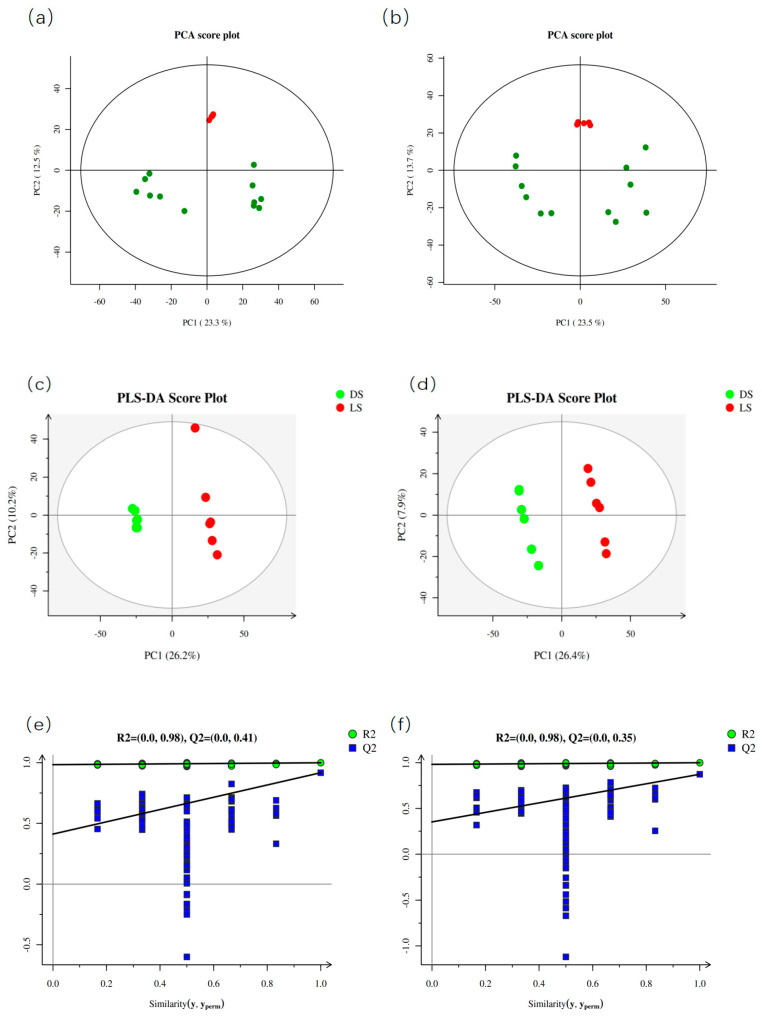
Multivariate analysis of the interroot soil metabolomic data of Radix et Rhizoma ginseng via LC–MS. (**a**,**b**): Principal component analysis (PCA) of the metabolomes of six continuous soil samples, six rotational crop samples, and quality control (QC) samples in positive (+) and negative (−) ion modes. (**c**,**d**): Partial least squares discriminant analysis (PLS-DA) in positive (+) and negative (−) ion modes, respectively. (**e**,**f**): Results of the permutation test for the PLS-DA model in positive (+) and negative (−) ion modes, respectively.

**Figure 2:**
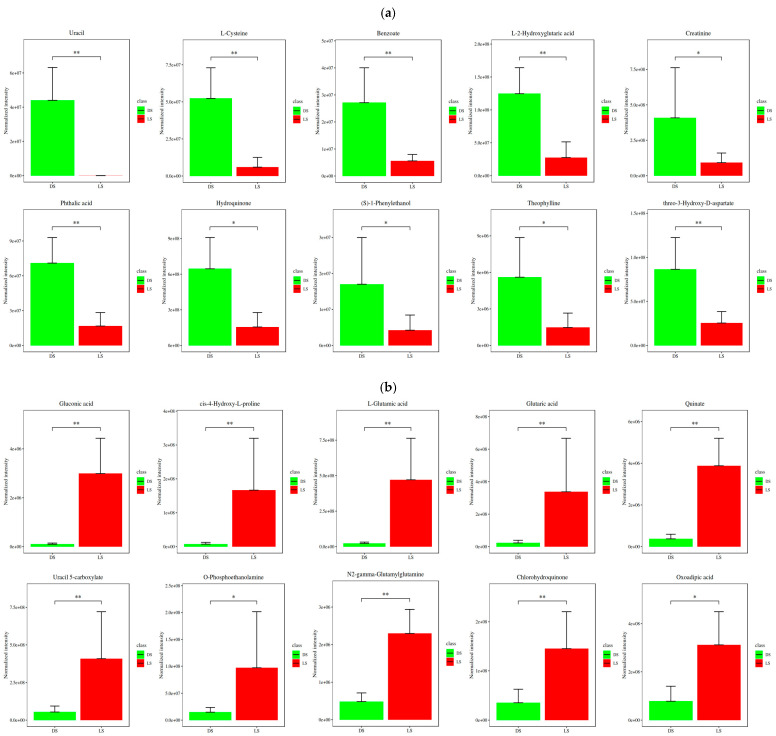

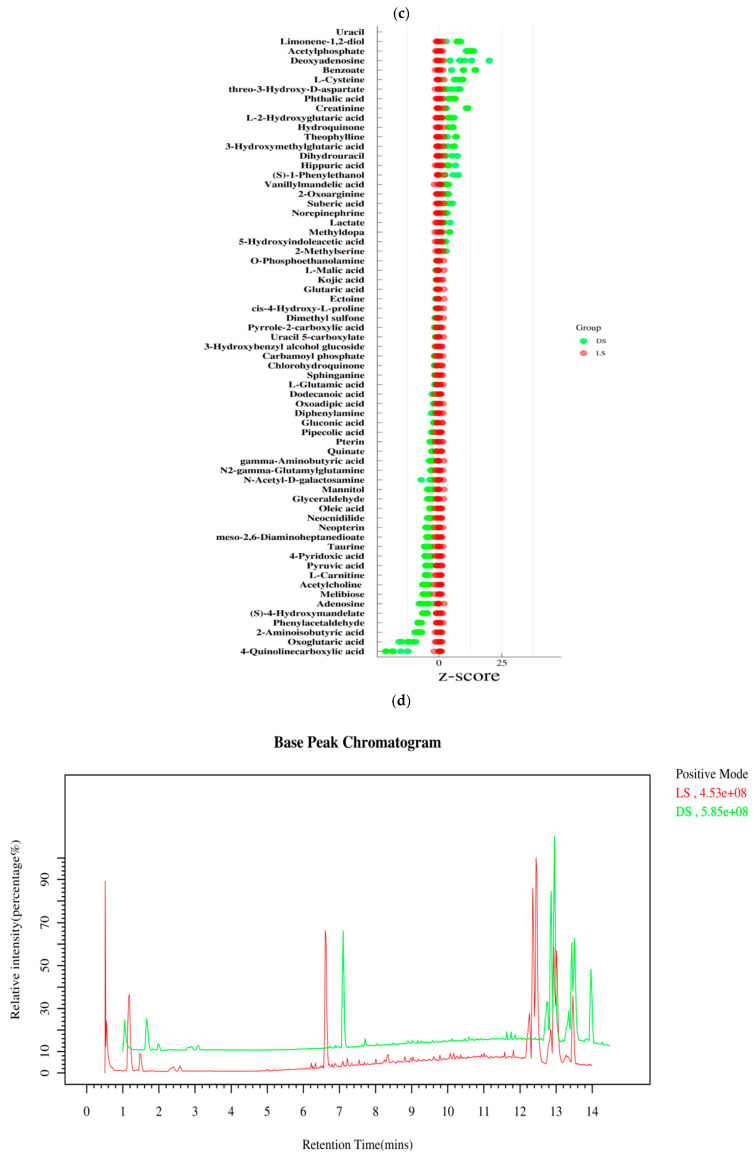

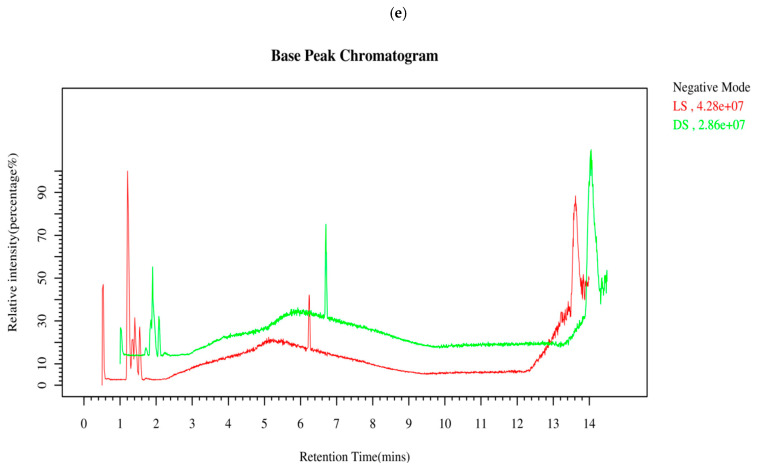
(**a**) Top 10 compounds with significantly upregulated differentially abundant metabolites. (**b**) Top 10 compounds with significantly downregulated differentially abundant metabolites. (**c**) Z-score plot. (**d**) Base peak chromatogram of typical soil samples in positive ion mode. (**e**) Base peak chromatogram of typical soil samples in negative ion mode. When *p* value < 0.05 and *p* value > 0.01, it is displayed as *; when *p* value < 0.01 and *p* value > 0.001, it is displayed as **.

Statistical analysis of 12 test samples was performed via PCA. The PCA results revealed significant differences in metabolites between the groups, with good separation. To obtain reliable and high-quality metabolomic data, quality control (QC) samples are typically used for quality control during detection. As can be seen from Figure 1a,b, the red circles corresponding to the quality control (QC) samples are closely clustered together: the smaller the differences between the QC samples, the greater the stability of the method and the better the quality of the data. The clustering of the data from the QC samples confirmed their reproducibility and reliability (Figure 1a,b). The analysis results indicated that the first two principal components (PCs) accounted for 35.8% of the total variance in positive ion mode (PC1 = 23.3%, PC2 = 12.5%) (Figure 2b) and 37.2% of the total variance in negative ion mode (PC1 = 23.5%, PC2 = 13.7%) (Figure 1a,b).

PLS-DA was performed on the metabolite accumulation levels to model the relationships that differentiated the sample groups. The PLS-DA model scores revealed that the DS and LS groups were different, which was consistent with the PCA results. The PLS-DA model had R^2^X values of 36.4% (+) and 34.2% (−), R^2^Y values of 99.9% (+) and 99.9% (−), and Q2 values of 91.7% (+) and 87.3% (−). This shows that the model has good predictive ability. To check whether the PLS-DA model is overfitted or not, 7-fold cross-validation and 200 response alignment tests were conducted to check the quality of the model, and the results proved that the model built is consistent with the real situation of the sample data and that the model is not overfitted (Figure 1e,f).

### 2.2. Screening for Differentially Expressed Metabolites

According to the screening criteria of VIP value > 1 and *p*-value < 0.05 in a *t*-test, metabolite screening was carried out. Eventually, 810 differentially expressed metabolites were obtained in positive ion mode (426 upregulated and 384 downregulated), and 861 differentially expressed metabolites were obtained in negative ion mode (183 upregulated and 678 downregulated). To screen for metabolites that change significantly under different physiological states or environmental conditions and to identify key metabolites associated with specific biological processes and environmental factors, fragment information obtained using MS/MS mode was further matched and annotated via standard databases such as the Human Metabolome Database (HMDB), Metlin, MassBank, LipidMaps, and mzCloud. Accurate information on metabolites was obtained, resulting in the identification of 232 secondary metabolites in total, of which 66 were differentially expressed (24 upregulated, 42 downregulated). The differentially abundant metabolites included mainly amino acids and their derivatives, nucleic acids, alcohols, organic acids, amines, fatty acids, purines, and sugars (Figure 2c). The fold differences in various metabolites between LS and DS were compared, with Figure 2a,b showing the top 20 differentially expressed metabolic components in terms of fold change. Compared with LS, compounds such as uracil, L-cysteine, benzoate, L-2-hydroxyglutaric acid, creatinine, phthalic acid, hydroquinone, (S)-1-phenylethanol, theophylline, threo-3-hydroxy-D-aspartate, oxoadipic acid, chlorohydroquinone, N2-gamma-glutamylglutamine, O-phosphoethanolamine, uracil 5-carboxylate, quinate, glutamic acid, L-glutamic acid, cis-4-hydroxy-L-proline, and gluconic acid were significantly different in DS. This finding is in line with numerous studies, where the rhizosphere soil metabolites predominantly consist of amino acids, saccharides, organic acids, and lipids. Li et al. found that with the extension of continuous cropping years, the relative abundances of lipid compounds, organic acids, and phenolic substances decreased, while the relative abundance of carbohydrates increased [38]. Yang et al. identified a variety of differential metabolites from the rhizosphere soil of *Codonopsis pilosula* in different continuous cropping years, including lipids, acids, sugars, other substances, esters, phenols, and amines [39]. This indicates that under different soil conditions, the metabolite species are comparable, yet their abundances vary [50,51].

### 2.3. Cluster Analysis of Differentially Abundant Metabolites

**Figure 3:**
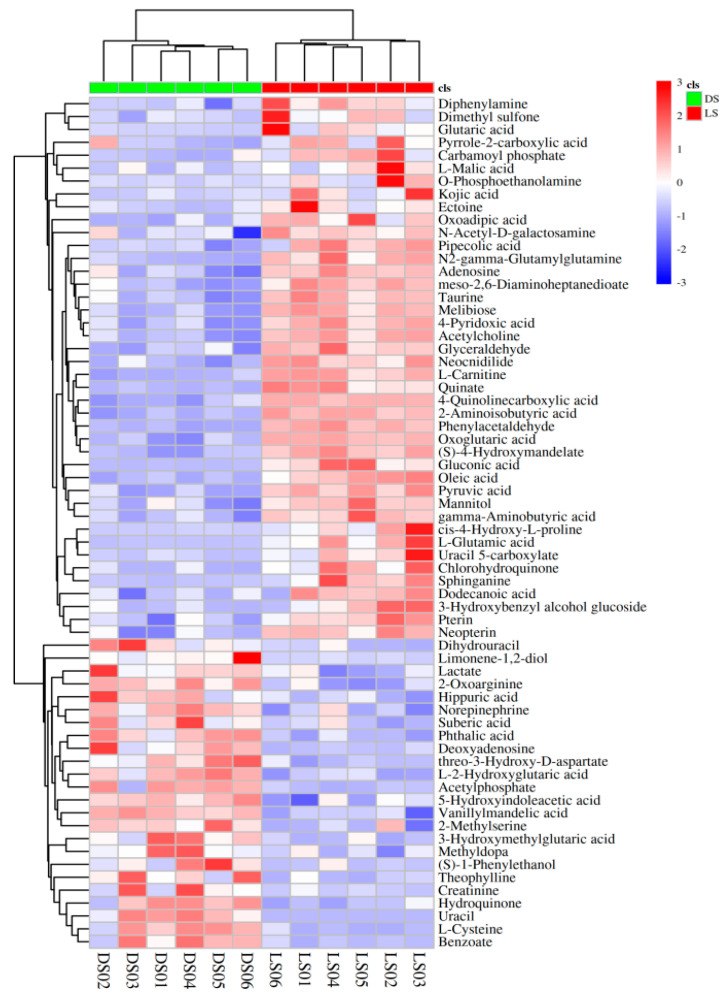
Heatmap of cluster analysis of differentially abundant metabolites in two interrooted soil samples of *Codonopsis pilosula* (rotational cropping (DS) and continuous cropping (LS)).

Hierarchical clustering analysis using Euclidean distance as the measure of dissimilarity was employed to visually present the differences in metabolite content in *Codonopsis pilosula* soil under different stubble conditions in the form of heatmaps. In the clustering analysis heatmap, each row represents the same differentially abundant metabolite, and each column represents the same *Codonopsis pilosula* sample. Different colors indicate different levels of metabolite content, with red representing high content and blue representing low content. Clustering analysis was performed on differentially abundant metabolites in *Codonopsis pilosula* soil under different crop rotations, and the results are represented in the form of heatmaps. Significant color distributions were observed, indicating distinct regions of high and low content expression in *Codonopsis pilosula* samples under different crop rotations. Comprehensive clustering analysis of *Codonopsis pilosula* samples (LS and DS) under different crop rotations identified a total of 66 differentially abundant metabolites, as shown in Figure 3. The heatmap shows the distribution of these differentially abundant metabolite contents in the two *Codonopsis pilosula* samples. The contents of metabolites such as benzoate, L-cysteine, creatinine, (S)-1-phenylethanol, and 2-methylserine were significantly greater in the DS samples than in the LS samples. However, the contents of metabolites such as quinate, glutamic acid, chlorohydroquinone, 4-quinoline carboxylic acid, and diphenylamine were significantly lower than those in the LS samples. The distribution of high-content (red) and low-content metabolites was clearly observed, with high-content metabolites (red) predominantly located at the bottom.

Amino acids and their derivatives are crucial metabolites in soil ecosystems. They directly supply nitrogen for plant growth and are involved in protein synthesis and other metabolic processes. Also, they serve as carbon and nitrogen sources for soil microorganisms, affecting microbial community structure and functions [37,52]. In the soil nitrogen cycle, their transformation and decomposition are closely related to nitrogen transformation, essential for maintaining nitrogen balance and availability [53]. They can interact with soil minerals and organic matter, improving soil structure, enhancing fertility, and potentially adjusting soil pH, thus being vital for soil ecosystem stability and plant growth. In crop rotation treatment, the metabolites aspartic acid and L-cysteine were significantly upregulated (Figure 3). L-cysteine, a proteinogenic amino acid, has various functions in biological systems [54]. In soil heavy metal stress, cysteine can participate in heavy metal chelation and detoxification. Conversely, in continuous cropping treatment, pipecolic acid was significantly enriched (Figure 3). Pipecolic acid can enhance defense responses and trigger coordinated induced immunity and systemic acquired resistance (SAR). Návarová et al. reported that defects in SAR, PTI, and ETI in ald1 mutant plants are related to a lack of Pip production, and exogenous Pip can compensate for these resistance deficiencies [55]. We speculate that stress from continuous cropping obstacles may lead to *Codonopsis pilosula* plants secreting large amounts of pipecolic acid metabolites, increasing plant resistance and alleviating the stress. Organic acids, as acidic compounds, can improve soil fertility by reacting with minerals to release insoluble nutrients, increasing nutrient availability. They also regulate soil pH, create a favorable environment for plant growth, and provide carbon sources and energy for soil microorganisms, driving changes in microbial community structure and function [56,57]. *Codonopsis pilosula* crop rotation increased the content of organic acids like phthalic acid and suberic acid (Figure 3). Hei et al. reported that applying more than 250 mg/kg exogenous organic acids (phthalic acid, palmitic acid, and a mixture) could increase soil microbial diversity [58]. Our previous studies showed that continuous cropping of *Codonopsis pilosula* leads to a decrease in soil microbial diversity and abundance, triggering continuous cropping obstacles. However, crop rotation with *Codonopsis pilosula* increases organic acid content and soil microbial diversity, enhancing plant immunity, improving soil structure, and increasing plant stress resistance. Continuous cropping of *Codonopsis pilosula* significantly increased the contents of organic acids such as oxoglutaric acid, pyruvic acid, glutaric acid, and quinate (Figure 3). Guo et al. found that quinate and its derivatives (e.g., chlorogenic acid) have astringent (bitter) and antibacterial properties and play crucial roles in plant defense [59]. The increased quinate content in continuously cropped *Codonopsis pilosula* can enhance plant defense ability, reducing the adverse effects of continuous cropping obstacles. In crop rotation treatment, the differentially abundant metabolites of purines and pyrimidines were significantly enriched. Purines and pyrimidines in the soil are important in ecosystems. They are essential components of nucleic acids and crucial for soil microorganism growth, reproduction, and genetic information transmission [60]. They participate in microbial metabolic processes, influencing microbial community structure and function. Additionally, they regulate the nitrogen cycle in the soil, providing a nitrogen source for plant growth [61]. Venter et al. reported that crop rotation significantly increased microorganism richness and diversity [27]. Liu et al. reported that in warm regions (MAT ≥ 8 °C), crop rotation significantly increased soil microbial biomass carbon (MBC) and nitrogen (MBN), while in cold regions (MAT < 8 °C), the bacterial Shannon diversity index significantly increased [62]. Wang et al. studied the rhizosphere soil of four crop rotation systems and found that compared with continuous cropping, crop rotation significantly altered the composition of rice rhizosphere metabolites, affecting soil microbial diversity and community composition [36]. *Codonopsis pilosula* crop rotation increases soil microbial diversity and functional diversity by increasing metabolite secretion such as purines and pyrimidines, thus improving the quality and yield of *Codonopsis pilosula*. The rhizosphere soil of continuously cropped *Codonopsis pilosula* accumulates amines, fatty acids, and carbohydrate metabolites. This may be due to reduced microbial diversity and abundance in the rhizosphere soil, decreasing the types and quantities of microorganisms involved in metabolite decomposition, thus reducing decomposition efficiency. It is also possible that continuous cropping of *Codonopsis pilosula* leads to adjustments in the plant’s physiological metabolic processes, reducing the demand for these metabolites and further exacerbating their accumulation in the soil.

### 2.4. Correlation Analysis of Metabolites

**Figure 4:**
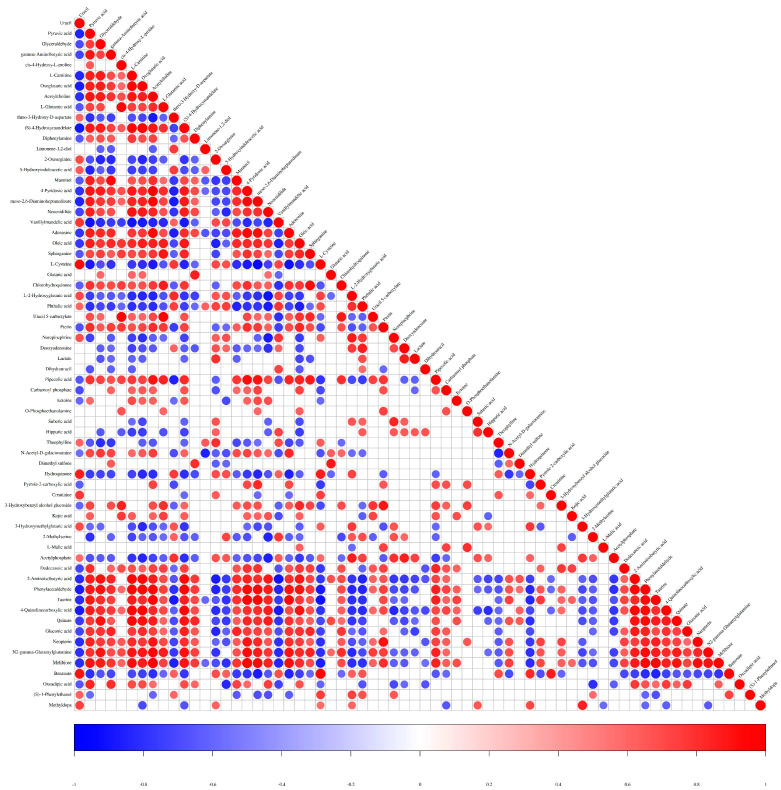
Metabolite–metabolite correlation analysis. Positive correlations are shown in red; negative correlations are shown in blue.

Differentially abundant metabolite association analysis involves studying the consistency of variation trends among metabolites by calculating the Pearson correlation coefficient or Spearman rank correlation coefficient between pairs of all metabolites to analyze their correlations. A total of 1272 correlations resulting in significant correlation coefficients (*p* < 0.01) were identified in the samples. Among these significant correlations, 730 were positively correlated, and 542 were negatively correlated (Figure 4). Significant positive correlations were detected between 4-pyridoxic acid and acetylcholine, melibiose and acetylcholine, (S)-4-hydroxymandelate and oxoglutaric acid, melibiose and 4-pyridoxic acid, and melibiose and taurine. In contrast, significant negative correlations were observed between vanillylmandelic acid and pyruvic acid, L-cysteine and adenosine, L-2-hydroxyglutaric acid and neocnidilide, taurine and L-cysteine, and L-cysteine and meso-2,6-diaminoheptanedioate. Notably, amino acids and their derivatives dominated the significant metabolite correlations, followed by organic acids, nucleosides, and sugars.

### 2.5. KEGG Pathway Analysis of Metabolites

**Figure 5:**
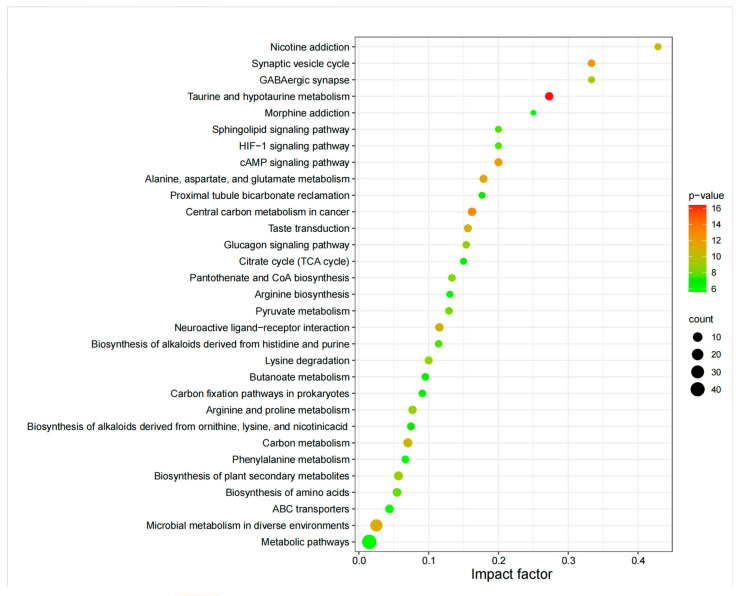
Top 30 enriched KEGG pathways from differentially accumulated metabolites (DAMs) identified between DS and LS.

To investigate the mechanisms underlying metabolite changes, the selected differentially abundant metabolites were subjected to KEGG pathway enrichment analysis. The results revealed the involvement of six pathways: cellular processes, environmental information processing, drug development, human diseases, genetic information processing, and metabolism. Notably, the metabolism category encompassed the most pathways, primarily arginine and proline metabolism, amino acid biosynthesis, biosynthesis of plant secondary metabolites, fatty acid degradation, metabolic pathways, and biosynthesis of alkaloids derived from histidine and purine. On the basis of functional enrichment analysis via the Kyoto Encyclopedia of Genes and Genomes (KEGG) database (Figure 5), the top 30 metabolic pathways were identified, encompassing 32 significantly differentially abundant metabolites (Table 1). These metabolic pathways include the biosynthesis of plant secondary metabolites, arginine and proline metabolism, lysine degradation, pantothenate and coenzyme A biosynthesis, pyruvate metabolism, amino acid biosynthesis, the HIF-1 signaling pathway, the sphingolipid signaling pathway, and the biosynthesis of alkaloids derived from histidine and purine. Compared with those in LS, the differentially abundant metabolites in DS were significantly enriched in amino acid metabolism (alanine, aspartate, and glutamate metabolism), carbon metabolism (citric acid cycle/tricarboxylic acid cycle), signaling pathways (cAMP signaling pathway), ABC transporters, phenylalanine metabolism, biosynthesis of plant secondary metabolites, and biosynthesis of secondary metabolites (biosynthesis of alkaloids derived from histidine and purine). In conclusion, we identified two key metabolic pathways related to continuous cropping and crop rotation: the biosynthesis of plant secondary metabolites and phenylalanine metabolism.

Research indicates that plants have specific mechanisms to resist stress [63]. In crop rotation treatment, abundances of amino acid metabolites like alanine, aspartate, and glutamate in rhizosphere metabolites increased significantly (Table 1). Aspartate is a precursor for amino acid synthesis. Sadak et al. reported that Asp treatment reduced ROS and increased compatible solutes and antioxidant activity in plants [64]. Foliar application of aspartate can also reduce cadmium uptake and stress in rice [65]. Enrichment of amino acid metabolic pathways is crucial for plant growth and stress resistance. The citric acid cycle in carbon metabolism is important for energy and material metabolism. Under stress, plants regulate their rate and flow. The cAMP signaling pathway in signal transduction regulates plant stress responses. When activated, it triggers intracellular responses to adapt to the environment [66]. ABC transporters are vital for plant metabolic balance and stress adaptation. In continuous cropping treatment, the abundance of related metabolites like taurine, L-aspartate, L-glutamate, and mannitol increased (Table 1). *Codonopsis pilosula* may enhance metabolite transport via ABC transporters to adapt to continuous cropping obstacles. ABC transporters take up nutrients and expel harmful metabolites, maintaining cell stability [67,68]. Metabolites from the phenylalanine metabolic pathway support rhizosphere microorganisms, enhancing plant adaptability [69]. Secondary metabolites like alkaloids, terpenoids, phenolic compounds, and flavonoids help plants adapt to abiotic stresses and maintain growth [70]. Phenolic compounds, especially flavonoids, scavenge free radicals and reduce oxidative stress [71]. Although not essential for growth, secondary metabolite biosynthetic pathways protect plants under stress [72].

## 3. Materials and Methods

### 3.1. Experimental Site and Sample Collection

The experimental site was located in Lasha Village, Lichuan Town, Dangchang County, Longnan City, Gansu Province. This area is situated in a temperate continental climate zone, positioned at 104°18′ E longitude and 34°20′ N latitude, with an altitude of 2224 m. The region experiences an average annual temperature of 8.3 °C, approximately 1976.5 h of sunshine per year, a frost-free period averaging 120 days annually, a solar radiation of 124.94 kcal/cm^2^, an average annual precipitation of 480 mm, and an average relative humidity of 64%. The climate is mild and humid, with sufficient sunlight and precipitation. The area typically experiences late warming in spring and a quick drop in temperature during autumn. The soil at the experimental site has the following basic nutrient characteristics: a pH value of 7.85, organic matter content of 12.79 g/kg, available phosphorus content of 22.43 mg/kg, nitrate nitrogen content of 16.53 mg/kg, ammonium nitrogen content of 12.64 mg/kg, and available potassium content of 165.72 mg/kg. The DS treatment involved preceding crops of astragalus and potato in the two years before the fields were planted with *Codonopsis pilosula*. The continuous cropping (LS) treatment involved the continuous planting of *Codonopsis pilosula* for two consecutive years.

During the sampling of the rhizosphere soil of *Codonopsis pilosula*, the shaking method was used. In late October 2023, *Codonopsis pilosula* plants were collected, ensuring that the root tissues remained intact and that any excess soil was removed. The soil adhering to the root tissues was gently brushed off and collected. The collected rhizosphere soil samples of *Codonopsis pilosula* were poured onto a flat table surface and mixed evenly. A straight line was then drawn diagonally across the sample. Two triangular samples were subsequently randomly selected and mixed. This process was continued until the remaining two apexes of the triangular samples approached the required sample amount by weight. Finally, the rhizosphere soil samples were preserved at a low temperature of −80 °C.

### 3.2. Metabolite Extraction

Sixty milligrams of sample was transferred into 2 mL centrifuge tubes. Next, 500 μL of methanol (USA) (precooled to −20 °C) and 500 μL of H_2_O (at 4 °C) were added, the mixture was vortexed for 30 s, and 100 mg of glass beads was added. The tubes were placed in liquid nitrogen for 5 min and thawed at room temperature. The tubes were subsequently placed into a tissue grinder and ground for 2 min at 55 Hz. The above step was repeated 3 times. The mixture was subsequently centrifuged at 4 °C for 10 min at 12,000 rpm, after which the supernatant was transferred to a new centrifuge tube. The samples were concentrated to dryness under vacuum. The samples were dissolved with 300 μL of 2-chlorobenzalanine (China) (4 ppm) methanol aqueous solution (1:1, at 4 °C), and the supernatant was filtered through a 0.22 μm membrane to obtain the prepared samples for LC–MS. Twenty microliters of each sample was taken for quality control (QC) analysis. The remaining samples were used for LC–MS detection.

### 3.3. LC–MS/MS Analysis

Chromatographic separation was conducted in a Thermo Vanquish (Thermo Fisher Scientific, Waltham, MA, USA) system equipped with an ACQUITY UPLC^®^ HSS T3 (150 × 2.1 mm, 1.8 µm, Waters, Milford, MA, USA) column which was maintained at a temperature of 40 °C. The temperature of the autosampler was set at 8 °C. The gradient elution of the analytes was carried out using 0.1% formic acid in water (A2) and 0.1% formic acid in acetonitrile (B2) (USA) or 5 mM ammonium formate in water (A3) (Japan) and acetonitrile (B3) at a flow rate of 0.25 mL/min. After equilibration, 2 microliters of each sample was injected. A linearly increasing gradient of solvent B2/B3 (*v*/*v*) was employed as follows: from 0 to 1 min, 2% B2/B3; from 1 to 9 min, 2% to 50% B2/B3; from 9 to 12 min, 50% to 98% B2/B3; from 12 to 13.5 min, 98% B2/B3; from 13.5 to 14 min, 98% to 2% B2/B3; and from 14 to 20 min, 2% B2-positive model (from 14 to 17 min, 2% B3-negative model).

The electrospray ionization multi-stage mass spectrometry (ESI-MSn) experiments were conducted on a Thermo Q Exactive (Waltham, MA, USA) mass spectrometer. This experiment adopted the data-dependent acquisition (DDA) mode. In the positive ion mode, the spray voltage was 3.5 kV, and in the negative ion mode, it was −2.5 kV. In this mode, fragmentation was performed on 10 (top N, N = 10) of the most intense peaks. The sheath and auxiliary gas were set at 30 and 10 arbitrary units, respectively. The capillary temperature was 325 °C. The analyzer carried out a full scan within the mass-to-charge ratio (*m*/*z*) range of 81–1000 with a mass resolution of 70,000. The tandem mass spectrometry (MS/MS) experiments adopted high-energy collision-induced dissociation (HCD) scanning, in which the normalized collision energy was set at 30 electron volts. The precursor ions (parent ions) selected for fragmentation were mainly based on their formation efficiency in the ion source, relative abundance, and structural relevance to the target analyte. Ions with high signal intensity and closely related to the chemical and structural characteristics of the target analyte were given priority. In addition, dynamic exclusion was used to remove some unwanted information from the MS/MS spectra.

### 3.4. Sample Data Preprocessing

The raw mass spectrometry files were converted to mzXML file format via the MSConvert tool in the ProteoWizard software package (v3.0.8789). Peak detection, filtering, and alignment were performed via the XCMS package in R to obtain a list of quantified substances. The parameters were set as follows: bw = 2, ppm = 15, peak width = c(5, 30), mzwid = 0.015, mzdiff = 0.01, and method = centWave’. Substance identification was conducted via public databases such as HMDB, MassBank, LipidMaps, mzCloud, KEGG, and a self-constructed substance library with the parameters set to ppm < 30. In this process, comprehensive analysis and identification were also carried out by combining multiple methods such as manual spectral analysis and literature verification. A series of statistical tests were employed to process the data more accurately. Firstly, variance analysis was carried out to evaluate the differences in metabolite contents among different sample groups. By calculating the F-value and *p*-value, it was determined which metabolites had significant differences between different groups. Then, *t*-tests were utilized to compare the contents of specific metabolites in two groups of samples and further screen out the metabolites with significant differences. Data calibration was accomplished by means of the LOESS signal correction approach that is founded on QC samples, with the aim of eradicating systematic errors. In the process of data quality control, substances possessing RSDs (Relative Standard Deviations) exceeding 30% in the QC samples were excluded.

Dimensionality reduction analysis of sample data was performed via PCA, PLS-DA, and OPLS-DA with the Roplal package in R. After data scaling, score plots, loading plots, and S-plots were generated to illustrate differences in metabolite composition among samples. Overfitting of the model was tested via permutation tests; R2X and R2Y represent the explained variance for the X and Y matrices, respectively, whereas Q2 reflects the predictive ability of the model. The closer these values are to 1, the better the model fit, and the more accurately the training set samples can be classified into their original groups. *p*-values were calculated via statistical tests, whereas VIP and FC values were computed via OPLS-DA to measure the impact and explanatory power of metabolite content on sample classification, aiding in the selection of marker metabolites. Metabolites are considered statistically significant when the *p*-value is <0.05 and the VIP > 1. Pearson correlation coefficients or Spearman rank correlation coefficients between all pairs of metabolites were calculated to analyze correlations, and a heatmap of differentially abundant metabolite associations was generated. Functional pathway enrichment and topological analysis of the selected differentially abundant metabolites were performed via the MetaboAnalystl10 software package (www.metaboanalyst.ca). The enriched pathways were visualized via the KEGG Mapper tool to browse the differentially abundant metabolites within the pathway maps.

## 4. Conclusions

In conclusion, this study analyzed in depth the metabolite differences in the rhizosphere soil of *Codonopsis pilosula* under rotation and continuous cropping through the nontargeted metabolomic method, revealing the metabolic mechanism of the continuous cropping obstacles of *Codonopsis pilosula*. The research results show that there are significant differences in the metabolite composition in the rhizosphere soil of *Codonopsis pilosula* under rotation and continuous cropping, especially in the content of amino acids and their derivatives, nucleic acids, organic acids, purine, and pyrimidine metabolites, as well as amines, fatty acids, and carbohydrate metabolites. These differential metabolites are mainly enriched in key metabolic pathways such as amino acid metabolism, carbon metabolism, signal pathways, ABC transporters, phenylalanine metabolism, and the biosynthesis of plant secondary metabolites. The results of this study emphasize the important role of metabolites in regulating the physical and chemical properties of soil and the structure of microbial communities, providing a theoretical basis and potential strategies for alleviating the continuous cropping obstacles of *Codonopsis pilosula* by regulating related metabolites and metabolic pathways. Future research should further explore the biosynthetic pathways of the differential metabolites in the rhizosphere soil of *Codonopsis pilosula* and their roles in growth and stress resistance. It is possible to study the influence of soil environmental factors (such as soil type, pH, nutrient availability, and microbial community), the growth stage of *Codonopsis pilosula*, and the planting methods on metabolites, explore the correlation between the metabolites in the roots of *Codonopsis pilosula* and the soil metabolites, and simultaneously analyze the two to determine whether there is a correlation or shared metabolic pathway, so as to better apply it to actual production and promote the healthy development of the *Codonopsis pilosula* industry.

## Figures and Tables

**Table 1:** Details of KEGG pathway enrichment.

KEGG Metabolic Pathway Name	Hits	−log(p)	Compounds	Pathway
Taurine and hypotaurine metabolism	6	16.388	C00022; C00025; C00026; C00097; C00227; C00245	map00430
Central carbon metabolism in cancer	6	13.074	C00022; C00025; C00026; C00097; C00149; C00186	map05230
cAMP signaling pathway	5	12.141	C00186; C00212; C00334; C00547; C01996	map04024
Synaptic vesicle cycle	4	12.114	C00025; C00334; C00547; C01996	map04721
Alanine, aspartate, and glutamate metabolism	5	11.55	C00022; C00025; C00026; C00169; C00334	map00250
Microbial metabolism in diverse environments	25	11.099	C00022; C00025; C00026; C00097; C00149; C00169; C00180; and 25 others	map01120
Neuroactive ligand–receptor interaction	6	11.03	C00025; C00212; C00245; C00334; C00547; C01996	map04080
Taste transduction	5	10.866	C00025; C00149; C00334; C00547; C01996	map04742
Carbon metabolism	8	10.617	C00022; C00025; C00026; C00097; C00149; C00169; C00227; C00257	map01200
Nicotine addiction	3	10.143	C00025; C00334; C01996	map05033
GABAergic synapse	3	9.2824	C00025; C00026; C00334	map04727
Biosynthesis of plant secondary metabolites	8	9.0946	C00022; C00025; C00026; C00097; C00149; C00408; C00712; C07130	map01060
Glucagon signaling pathway	4	8.8181	C00022; C00026; C00149; C00186	map04922
Arginine and proline metabolism	6	8.7114	C00022; C00025; C00334; C00791; C03771; C05942	map00330
Lysine degradation	5	8.6606	C00026; C00322; C00408; C00489; C03196	map00310
Pantothenate and CoA biosynthesis	4	8.2442	C00022; C00097; C00106; C00429	map00770
Pyruvate metabolism	4	8.114	C00022; C00149; C00186; C00227	map00620
Biosynthesis of amino acids	7	7.8525	C00022; C00025; C00026; C00097; C00169; C00322; C00680	map01230
HIF-1 signaling pathway	3	7.6388	C00022; C00026; C00186	map04066
Sphingolipid signaling pathway	3	7.6388	C00212; C00346; C00836	map04071
Biosynthesis of alkaloids derived from histidine and purine	4	7.6368	C00022; C00026; C00149; C07130	map01065
Biosynthesis of alkaloids derived from ornithine, lysine, and nicotinic acid	5	7.2832	C00022; C00025; C00026; C00149; C00408	map01064
Proximal tubule bicarbonate reclamation	3	7.2522	C00025; C00026; C00149	map04964
Butanoate metabolism	4	6.933	C00022; C00025; C00026; C00334	map00650
Citrate cycle (TCA cycle)	3	6.7584	C00022; C00026; C00149	map00020
Carbon fixation pathways in prokaryotes	4	6.756	C00022; C00026; C00149; C00227	map00720
Arginine biosynthesis	3	6.3407	C00025; C00026; C00169	map00220
Metabolic pathways	42	6.0254	C00022; C00025; C00026; C00097; C00106; C00149; C00169; and 42 others	map01100
Morphine addiction	2	5.8055	C00212; C00334	map05032

## Data Availability

The data presented in this study are available upon request from the corresponding author.
